# Rimonabant Kills Colon Cancer Stem Cells without Inducing Toxicity in Normal Colon Organoids

**DOI:** 10.3389/fphar.2017.00949

**Published:** 2018-01-04

**Authors:** Donatella Fiore, Prashanthi Ramesh, Maria C. Proto, Chiara Piscopo, Silvia Franceschelli, Serena Anzelmo, Jan P. Medema, Maurizio Bifulco, Patrizia Gazzerro

**Affiliations:** ^1^Department of Pharmacy, University of Salerno, Fisciano, Italy; ^2^Laboratory of Experimental Oncology and Radiobiology, Center for Experimental and Molecular Medicine, Academisch Medisch Centrum, University of Amsterdam, Amsterdam, Netherlands; ^3^Department of Molecular Medicine and Medical Biotechnologies, University of Naples “Federico II”, Naples, Italy

**Keywords:** colon cancer, colon cancer stem cells, Rimonabant, cannabinoids, 3D cultures, Wnt/β-Catenin

## Abstract

Colorectal cancer (CRC), like other tumor types, is a highly heterogeneous disease. Within the tumor bulk, intra-tumoral heterogeneity is also ascribable to Cancer Stem Cells (CSCs) subpopulation, characterized by high chemoresistance and the unique ability to retain tumorigenic potential, thus associated to tumor recurrence. High dynamic plasticity of CSCs, makes the development of winning therapeutic strategies even more complex to completely eradicate tumor fuel. Rimonabant, originally synthesized as antagonist/inverse agonist of Cannabinoid Receptor 1, is able to inactivate Wnt signaling, both *in vitro* and *in vivo*, in CRC models, through inhibition of p300-histone acetyltransferase activity. Since Wnt/β-Catenin pathway is the main player underlying CSCs dynamic, this finding candidates Rimonabant as potential modulator of cancer stemness, in CRC. In this work, using established 3D cultures of primary colon CSCs, taking into account the tumor heterogeneity through monitoring of Wnt activity, we demonstrated that Rimonabant was able to reduces both tumor differentiated cells and colon CSCs proliferation and to control their survival in long term cultures. Interestingly, in *ex vivo* model of wild type human organoids, retaining both architecture and heterogeneity of original tissue, Rimonabant showed no toxicity against cells from healthy colon epithelium, suggesting its potential selectivity toward cancer cells. Overall, results from this work provided new insights on anti-tumor efficacy of Rimonabant, strongly suggesting that it could be a novel lead compound for CRC treatment.

## Introduction

Colorectal cancer (CRC) development is typically associated with a stepwise accumulation of specific genetic alterations, collectively known as “adenoma-carcinoma sequence,” characterized by pathologic alterations, ranging from microscopic lesions, such as Aberrant Crypt Foci (ACF), to metastatic phenotypes. However, in the last decades has been highlighted that adenoma-carcinoma sequence arises in a limited portion of CRC cases (about 50–60%) (Fearon, 2011), suggesting a higher complexity, which results in high heterogeneity. Genetic, epigenetic and environmental factors are drivers of inter- and intra-tumoral heterogeneity and the cancer stem cells (CSCs) theory increased complexity, testifying that even intra-clonal heterogeneity exists (Zeuner et al., 2014; Punt et al., 2016).

Cancer stem cells are dynamic subset of cells within tumor bulk, likely derived from transformation of stem cells, characterized by indefinite self-renewal ability and thus able to retain tumorigenic potential, supporting hierarchical organization of CRC. On the other hand, it is now clear that tumorigenesis can be considered a plastic process in which also non-CSCs differentiated cells, depending on several intrinsic and extrinsic factors, can acquire self-renewal ability and thus became CSCs (Prasetyanti and Medema, 2017). In addition to loss of differentiation and self-renewal ability, in high malignancy, chemoresistance and thus relapses onset are substantially ascribable to CSCs (Colak and Medema, 2014). As such, these observations allowed development of novel strategies targeting CSC or at least restraining their survival.

Wnt/β-Catenin pathway is a highly conserved signaling, known to exert a key role in the most part of cancer-related processes (Anastas and Moon, 2013; Deitrick and Pruitt, 2016). Inactivating mutations of APC (Adenomatous Polyposis Coli) tumor suppressor gene and activating mutation of β-Catenin gene, are associated with both familial syndromes (FAP) and sporadic CRC (Fearon, 2011). Together with several mutations throughout signaling transduction, these alterations lead to Wnt hyperactivation and, then, enhanced β-Catenin nuclear activity downstream canonical signaling (Clevers and Nusse, 2012). Vermeulen et al. (2010) demonstrated that in spheroidal cultures of Colon CSCs, Wnt activity appears to be heterogeneous and inversely correlated with differentiation. In this scenario, only cells with high Wnt activity can be defined CSCs with tumorigenic ability. However, they also demonstrated that differentiated tumor cells are influenced by extrinsic micro-environmental factors to acquire high Wnt activity, and then become CSCs, suggesting their complex and dynamic regulation (Vermeulen et al., 2010).

In recent years, research and development of drugs able to specifically target Wnt/β-Catenin in CSCs and to act at multiple level of WNT-mediated signal transduction, become a priority (Song et al., 2015). Porcupine inhibitor LGK974 and Frizzled (Fzd) receptor inhibitor, OMP-18R5, are under clinical investigation as compounds able to control Wnt signaling through plasma membrane (clinical trial NCT01351103 and NCT01345201 respectively). Sulindac and Celecoxib, two non-steroidal anti-inflammatory drugs (NSAIDs), are able to inhibit cytoplasmic Wnt signaling acting as Dvl and β-Catenin inhibitors respectively (Song et al., 2015; Takebe et al., 2015). Beyond TCF/LEF transcription factor, the complex transcriptional machinery regulated by β-Catenin on Wnt Responsive Elements (WRE) includes, among other proteins, p300 (Kat3b) and CREB-binding protein (CBP or Kat3a) co-activators. p300 and CBP are two high-related proteins, able to coordinate chromatin remodeling complex through their histone lysine acetyltransferases (HAT) catalytic activity (Mosimann et al., 2009). Several reports demonstrated that both CBP-selective (ICG-001 and PRI-724) and CBP/p300 non-selective (C646) inhibitors are able to exert anti-tumor effects in CRC, counteracting β-Catenin binding and its transcriptional activity on WRE (Gaddis et al., 2015; Bordonaro and Lazarova, 2016).

Anti-tumor action of cannabinoids in CRC was strongly supported by several authors. The Endocannabinoid (EC) system role in the progression of CRC has been analyzed *in vivo* in the mouse model of azoxymethane-induced colon carcinogenesis, where cannabinoids-mediated reduction of precancerous lesions in the mouse colon was found (Izzo et al., 2008; Santoro et al., 2009). In CRC cells, agonists and antagonists of both cannabinoid receptors, CB1 and CB2, showed anti-tumor action through induction of cell death with different mechanisms ranging from apoptosis to mitotic catastrophe (Greenhough et al., 2007; Cianchi et al., 2008; Izzo et al., 2008; Santoro et al., 2009).

In our recent work, we found that Rimonabant, originally synthetized as CB1 antagonist/inverse agonist, exerts its anti-tumor effects through inhibition of Wnt/β-Catenin-mediated signaling. In particular, in CRC models, Rimonabant inhibits signal transduction through plasma membrane, induces β-Catenin degradation and reduces its nuclear translocation, both *in vitro* and *in vivo*. Moreover, our results depicted, for the first time, a novel epigenetic mechanism, CB1-independent, of Rimonabant. Through *in silico* tools, substantially confirmed by Surface Plasmonic Resonance assay, we found a direct interaction between Rimonabant and p300-HAT domain. As such, inhibition of HAT activity, together with upstream inhibition of Wnt/β-Catenin signal, results in downregulation of Wnt-modulated genes: Cyclin D1, c-Myc and COX2 (Proto et al., 2017). These findings candidate Rimonabant as potential compound able to control colon cancer stemness.

Based on previous results, in this work, using established 3D *in vitro* model of primary CSCs described by Vermeulen et al. (2010), we investigated for the first time the ability to control CSCs proliferation and spreading exerted by Rimonabant used as single agent and in combination with both Oxaliplatin and 5-Fluorouracil (5FU). Moreover, we obtained preliminary insights on the Rimonabant effects in healthy colon epithelium using *ex vivo* model of wild type human organoids.

## Materials and Methods

### General Materials

Rimonabant (also referred as SR141716) was kindly donated by Sanofi-Aventis (Montpellier, France). It was dissolved in DMSO and added to cells cultures at the indicated concentrations. 5-Fluorouracil and Oxaliplatin were purchased from Sigma–Aldrich (Dorset, United Kingdom).

Anti-phospho LRP6 (Ser1490), anti-LRP6, anti-GAPDH and secondary HRP-linked goat anti-mouse or goat anti-rabbit IgG were purchased from Cell Signaling Technology. Anti-CD44, anti-EpCAM, anti-CD133, anti-CB1, anti-Lgr5 and anti-β-Catenin were from Abcam.

### Cell Cultures and Treatments

Human CRC cells HCT116 and DLD1 were obtained from the Interlab Cell Line Collection (IST, Genoa, Italy) and routinely grown in McCoy’s 5A and RPMI medium, respectively, supplemented with 10% fetal bovine serum (FBS), 2 mM glutamine, 100 U/ml penicillin, 100 μg/ml streptomycin, in monolayer culture and incubated at 37°C in a humidified atmosphere containing 5% CO2.

Primary Colorectal Cancer Stem Cell line, GTG7, was kindly provided by Prof. Jan Paul Medema [Academisch Medisch Centrum (AMC), Centre for Experimental and Molecular Medicine (CEMM), Laboratory of Experimental Oncology and Radiobiology (LEXOR), University of Amsterdam] and Prof. Giorgio Stassi (University of Palermo, Italy) and obtained from patients as described in Prasetyanti et al. (2013). GTG7 cells were cultured and propagated as spheroids in ultra-low adherent supports, in CSC medium (Advanced DMEM-F12) supplemented with: N2 supplement (Gibco), 6 mg/ml glucose, 5 mM HEPES, 2 mM L-glutamine, 4 μg/ml heparin, 50 ng/ml Epidermal Growth Factor (EGF) and 10 ng/ml basic Fibroblast Growth Factor (bFGF). GTG7 spheroids are established cells containing a TCF/LEF-driven GFP reporter for Wnt-signaling activity (Wnt-TOP-GFP), as described in Vermeulen et al. (2010). In this system, the 10% highest expressing Wnt-GFP (TOP-GFP^high^ or Wnt^high^) represents CSCs, while the 10% lowest (TOP-GFP^low^ or Wnt^low^) identificates differentiated tumor cells.

### Cell Viability and Drug Combination Analysis

HCT116 cells were exposed to various concentrations of compounds for the time points showed in the figures and to evaluate cells viability, colorimetric MTT metabolic activity assay was used. To this aim, MTT stock solution (5 mg/ml in PBS, Sigma) was added to each well and incubated for 4 h at 37°C in humidified CO2. At the end of the incubation, the medium was removed and the formazan crystals were solubilized with acidic isopropanol (0.1 N HCl in absolute isopropanol). MTT conversion to formazan by metabolically viable cells was monitored by spectrophotometer at an optical density of 570 nm. Each data point represents the average of three separate experiments in triplicate.

The relative contribution of Rimonabant and 5FU to the anti-proliferative effect in HCT116 cells and establishment of pharmacological interaction type, were calculated using Compusyn, dedicated software based on Chou-Talalay method (Chou and Talalay, 1984). Through median-effect equation, that is the common-link for single and multiple ligand interactions, this software allow to calculation of Combination Index (CI) to define synergism (CI < 1), additive effect (CI = 1) and antagonism (CI > 1). Moreover, Dose reduction index (DRI) represents the measure of how much the dose of each drug in a combination may be reduced at a given effect level compared with the doses of each drug alone (Chou, 2010). Assessment of drug interactions was performed calculating CI after treatment for 24 h with 1:2.5 Rimonabant/5FU in combination and as single drugs. The treatments were performed at least in triplicate, in three independent experiments.

### Western Blot Analysis

Total protein extracts were obtained through lysis in buffer A (50 mM Tris–HCl pH8.0 buffer containing 150 mM NaCl, 1% Nonidet P-40, 2 mg/ml aprotinin, 1 mg/ml pepstatin, 2 mg/ml leupeptin, 1 mM Na_3_VO_4_). Protein concentration was determined by the Bradford assay using bovine serum albumin as standard. 10–30 μg of proteins were loaded and subjected to 8–12% SDS-PAGE, under reducing conditions. Gels were electroblotted into nitrocellulose membranes (Millipore Co) and filters were probed with the indicated primary anti-bodies.

### Cytofluorimetric Analysis of CSCs Markers, Caspase 3 Activation and Nicoletti Assay

To measure cell death at single cell level in Wnt^high^ (CSCs) and Wnt^low^ (differentiated colon cancer cells), cytofluorimetric analysis of caspase 3 activation was performed. GTG7 spheroids were dissociated as single cells and seeded in triplicate, in 12 multiwell plate (50000 cells/well) in adherent condition. After overnight adherence, cells were exposed to drugs, at indicated doses and time points. At the end of treatments, GTG7 were collected as single cells using Trypsin-EDTA and washed with CSC medium. Caspase 3 activity was measured using CaspGlow active staining kit (Red-DEVD-FMK), according to manufacturer’s instructions (BioVision). Briefly, after collection and washing, cells were pelleted and stained with RED-DEVD-FMK substrate for 1 h at 37°C. Subsequently, cells were washed twice with wash buffer and flow cytometry was performed with FACS Canto (BD bioscience). Cell death was measured analyzing PE intensity in 10% gated Wnt-GFP^high^ and 10% Wnt-GFP^low^ cells.

To measure DNA fragmentation status, Nicoletti assay was performed. Cells were seeded and collected as described above, then suspended in Nicoletti buffer (0.1% sodium citrate (w/v) and 0.1% Triton X-100 (v/v) in deionized water pH 7.4, supplemented with 50 μg/ml propidium iodide before use). After incubation at 4°C, PI staining of nuclei was measured using flow cytometry (FACS Canto).

Analysis of CSCs markers was performed incubating HCT116 and GTG7 cells with CD133/1 (AC133)-PE conjugated anti-body (Miltenyi biotec) and CD44-APC conjugated anti-body (Biolegend^®^), according to manufacturer’s instructions. For GTG7 cells analysis was carried out as described previously, gating on about 10% Wnt-GFP^low^ and 10% Wnt-GFP^high^ cells.

All experiments were performed in triplicate and repeated at least three independent times. Data were analyzed with FlowJo^®^ software (BDIS).

### Confocal Microscopy

GTG7 were grown in adhesion conditions on slides in 24 well plates. After treatment, cells were fixed in paraformaldehyde (PFA, 3,7% v/v in PBS) for 15 min, washed and permeabilized in Tryton X-100 (0,1% v/v in PBS) for 10 min. Then, cells were blocked with 4% Bovine Serum Albumin (BSA) for 1 h at room temperature and incubated with anti-β-Catenin, anti GPCR-GPR49 (Lgr5) and anti GFP primary anti-bodies, at 4° C overnight. Immunofluorescence staining was obtained by incubating for 1 h with Alexa Fluor^®^ 488 (FITC-conjugated) and Alexa Fluor^®^ 647 (Cy5-conjugated) secondary anti-bodies. Nuclei were stained with Hoechst 33342 dye (Thermo Fisher Scientific). The slides were mounted using mowiol mounting medium; a Zeiss LSM 510 Laser Scanning Microscope (Carl Zeiss MicroImaging GmbH, Jena, Germany) for data acquisition was used. Samples were vertically scanned from the bottom of the coverslip with a total depth of 5 μm and a Plan-Apochromat oil-immersion objective (magnification 63X^∗^1.7; 1.40 NA).

### Cell Survival Assay in GTG7

Spheroids cultures of CSCs were dissociated and seeded as single cells in 12 multiwell plate (50000 cells/well). After overnight adherence, GTG7 were treated with Rimonabant as in cell death assay. At the end of treatments, they were collected after exposure with trypsin-EDTA and pelleted, to remove the compound from the medium. For each treatment points, 20000 cells were distributed in triplicate in 96 ultralow adherent multiwell (Corning). Clones development, and then cell survival of CSCs, was measured at different time points, starting from day 0 (corresponding to 24 h treatments) until day 13, through colorimetric viability assay, by adding 20 μl/well of Cell Titer Blue (CTB) reagent (Promega). After 4 h of incubation, viability was monitored by spectrophotometer (560_Ex_/590_Em_ nm). The IC50 values for GTG7 were calculated at the end of 24 h of treatment with increasing doses of Rimonabant (see **Supplementary Table S3**). Each data point represents the average of three separate experiments in triplicate. Statistical analysis was performed with GraphPad Prism software©.

### Normal Colon Human Organoids Cultures and Clonogenic Assay

Normal colon human organoids culture was kindly provided by Prof. Jan Paul Medema (AMC, University of Amsterdam), obtained as described in Sato et al. (2011). The crypts were cultured in matrigel, in Normal colon culture medium: advanced DMEM/F12, supplemented with N2 and B27 supplement, Pen/Strep, gentamycin, amphotericin B, 2 mM GlutaMax-1, 10 mM HEPES, 1 mM *N*-acety-L-cysteine (Sigma), 10 nM [Leu15]-gastrin I (Sigma), 10 mM nicotinamide (Sigma), 500 nM A83-01 (Tocris), 3 μM SB202190 (Sigma), 50% WNT3A conditioned medium, 50 ng/ml h-EGF, 20% RSPO1 conditioned medium, 10% Noggin conditioned medium, 10 nM PGE2 (Santa Cruz Biotechnology).

For DNA fragmentation analysis, matrigel was mechanically destroyed and crypts were resuspended in Cell Recovery solution (BD bioscience). After incubation on ice, Nicoletti assay was performed as previously described.

To assess Rimonabant effects on normal colon organoids clonogenicity, 50–100 crypts were diluted in matrigel and seeded in 24 multiwell plate. After overnight incubation in normal colon medium, organoids were counted under microscope and then treated for 24 h with Rimonabant 10, 15 and 20 μM. After treatments, matrigel was mechanically destroyed, organoids were collected and pelleted to remove Rimonabant. Then, for each replicate all organoids were diluted in new matrigel and seeded again in 12 multiwell plate, with fresh medium. After 7 days, vital crypts were counted under microscope. Each data point represents the average of two separate experiments in duplicate.

### Statistical Analysis

Data obtained from multiple experiments were calculated as means ± SD and analyzed for statistical significance by using the two-tailed Student *t*-test, 1- or 2-way ANOVA for independent groups, with the Tukey or Bonferroni correction for multiple comparisons. Values of *P* < 0.05 were considered statistically significant.

## Results

### Rimonabant Controls Cancer Stemness and Chemoresistance in Human CRC Cells

A growing number of evidence indicates that Wnt hyperactivity is closely related to stem-like phenotype and a large numbers of markers helps in the identification, isolation and targeting of CSCs (Kemper et al., 2010; Vermeulen et al., 2010).

**FIGURE 1:**
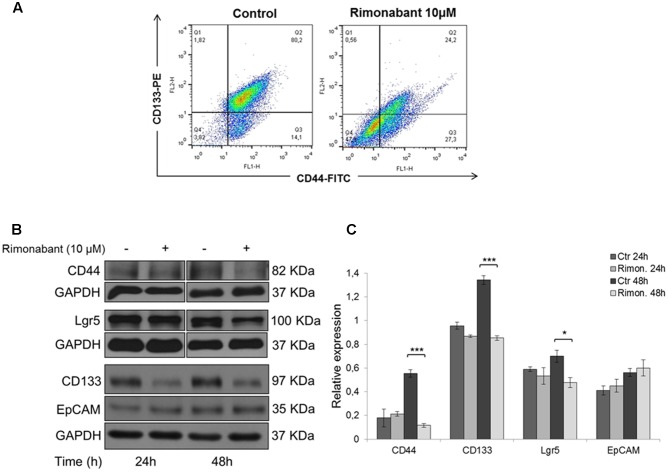
**(A)** FACS analysis of CD133-PE/CD44-FITC double stained HCT116 cells after treatment for 48 h with Rimonabant 10 μM. **(B)** Representative Western blot analysis of CD44, Lgr5, CD133 and EpCAM in total protein lysates from HCT116 cells. GAPDH was used as loading control. **(C)** Densitometric analysis of CD44, Lgr5, CD133 and EpCAM normalized vs. GAPDH. Results are expressed as means ± SD of 3 independent experiments performed at least in duplicate (^∗^*p* < 0.05, ^∗∗∗^*p* < 0,001 vs. control).

Our previous results showed that Rimonabant is able to inhibit Wnt/β-Catenin signaling through a mechanism involving p300/Kat3b-HAT inhibition (Proto et al., 2017). Using HCT116 cells as preliminary model to establish if Rimonabant can control cancer stemness, FACS analysis of CD133/CD44 double positive population was performed. After 48 h, Rimonabant significantly reduces CD133/CD44 double positive HCT116 cells (**Figure 1A**). Rimonabant-mediated reduction of CD133 and CD44 expression has been also confirmed through western blot analysis. However, Rimonabant does not modulate the expression of another well know CSCs marker, EpCAM (**Figures 1B,C**). As for CD44, Lgr5 is a Wnt-regulated gene, a CSC marker required for the maintenance of CRC-derived liver metastasis (Melo et al., 2017) and specifically associated to 5FU chemoresistance in colon cancer patients (Hsu et al., 2013). In line with the observed reduction of CD133 and CD44 expression, in HCT116 cells Rimonabant downregulates Lgr5 (**Figures 1B,C**), suggesting a potential role of the compound in the control of cancer stemness.

To strengthen our hypothesis, we analyzed Rimonabant-mediated modulation of CSCs markers in DLD1 human CRC cell line. The efficacy of Rimonabant and its ability to modulate Wnt signaling in DLD1 cells, was previously reported by our group (Santoro et al., 2009; Gazzerro et al., 2010; Proto et al., 2017). In DLD1 cells, Rimonabant strongly inhibits CD133 expression starting from 24 h of treatment while after 48h the expression of both CD133 and CD44 markers results significantly reduced, reinforcing our hypothesis. However, Lgr5 expression was not affected by Rimonabant treatment, in DLD1 cells (see **Supplementary Figure S1A**).

Finally, accordingly to CD133 and CD44 reduction, after 48 h of treatment, in both HCT116 and DLD1 cells Rimonabant significantly reduces β-Catenin expression (see **Supplementary Figure S1B**), as we previously reported. Indeed, in Proto et al. (2017), we demonstrated that as early effect, increase of phosphorylated (Ser33/37) β-Catenin occurs after treatment with Rimonabant. The increase of β-Catenin phosphorylation led to its proteasome degradation and, thus, to reduction of total expression and inhibition of its nuclear translocation and activity (Proto et al., 2017).

**FIGURE 2:**
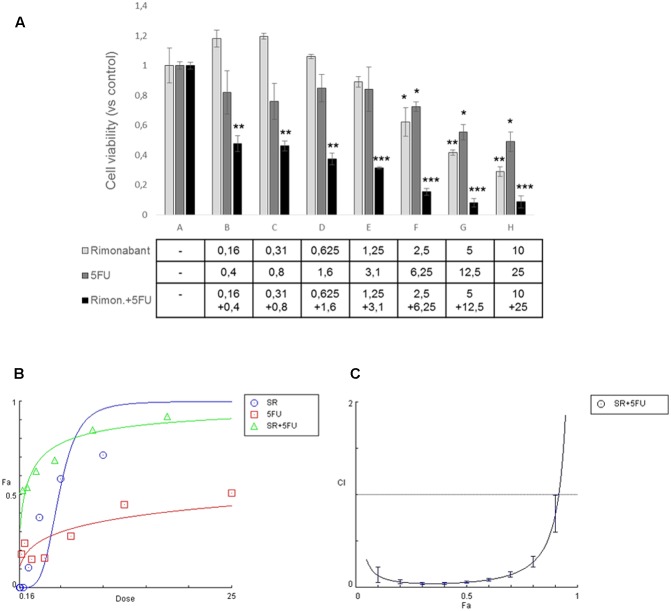
**(A)** MTT assay performed in HCT116 cells treated with indicated concentrations of Rimonabant (μM) or 5FU (μM) alone or in combination; data are expressed as means ± SD of 3 independent experiments performed in triplicate and reported as ratio vs. the vehicle control (^∗^*p* < 0.05, ^∗∗^*p* < 0.01, ^∗∗∗^*p* < 0.001 vs. control). **(B)** Representative dose-effect curve performed at least twice in triplicate with CompuSyn software and showing the dose of the drug *vs.* the fraction of cells affected/killed (Fa) by Rimonabant (SR) or 5FU used alone or combined at constant molar ratio 1:2.5. **(C)** Combination Index plot (Fa-CI plot) computed by Compusyn software from the affected fraction (Fa) obtained at each dose of the drugs used in combination, at constant molar ratio 1:2.5. Representative experiment (carried out at least twice) is reported.

Since we showed that Rimonabant reduces Lgr5 expression in HCT116, to evaluate if it could improve the anti-tumor effects of 5-Fluorouracil, one of the most used chemotherapeutics in CRC treatment, MTT assay was performed in HCT116 cells treated for 24 h with the compound used alone or combined with 5FU. Specifically, Rimonabant (range 0.16 – 10 μM) used at constant molar ratio (1:2.5) with 5FU (range 0.4 – 25 μM) induced a significant cytotoxicity higher than that observed for the single doses of both Rimonabant and 5FU used alone (**Figures 2A,B**). Checking for multi-drug interactions, the analysis of data performed with Compusyn software, revealed a Combination Index ≤ 1, starting from 0.16 and 0.4 μM and until 5 μM and 12.5 μM of Rimonabant and 5FU, respectively, suggesting a synergistic interaction (**Figure 2C** and **Supplementary Table S1**). The Dose Reduction Index obtained for both compounds strongly highlighted the edge offered by the combined use in HCT116 cell lines (see **Supplementary Table S2**). The IC50 values of HCT116 to Rimonabant and 5FU are reported in **Supplementary Table S3**.

### Rimonabant Induces Cell Death in Primary Colon CSC Line GTG7

**FIGURE 3:**
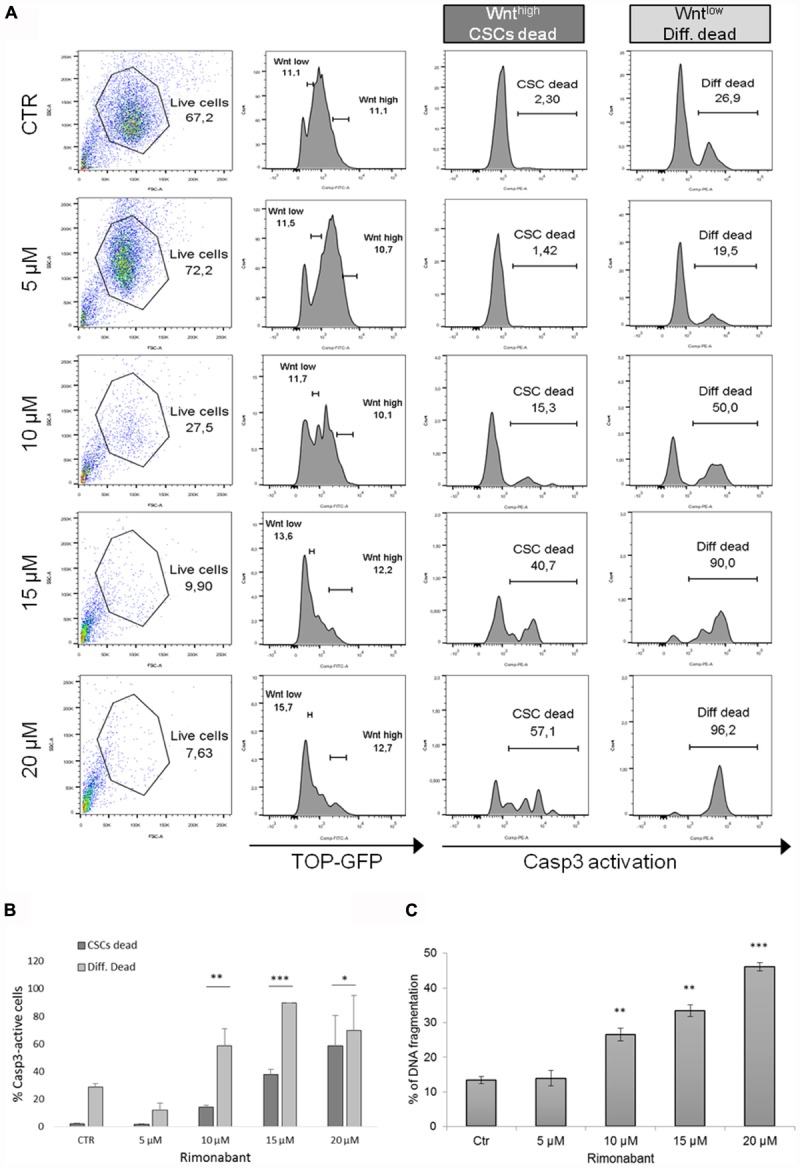
**(A)** Representative histograms and **(B)** bar histogram of Caspase 3 activation in GTG7 cells treated for 24 h with Rimonabant or vehicle alone at the indicated doses. Caspase 3 (PE quantification) was analyzed gating on about 10% of TOP-GFP^high^ (Wnt^high^) cells, corresponding to CSCs, and on about 10% TOP-GFP^low^ (Wnt^low^) cells, corresponding to differentiated tumor cells. **(C)** DNA fragmentation amount in PI-stained GTG7 cells, treated for 24 h with Rimonabant or vehicle alone at the indicated doses. Data are expressed as mean ± SD of at least three independent experiments in duplicate (^∗^*p* < 0.05, ^∗∗^*p* < 0.01, ^∗∗∗^*p* < 0.005 vs. control).

**FIGURE 4:**
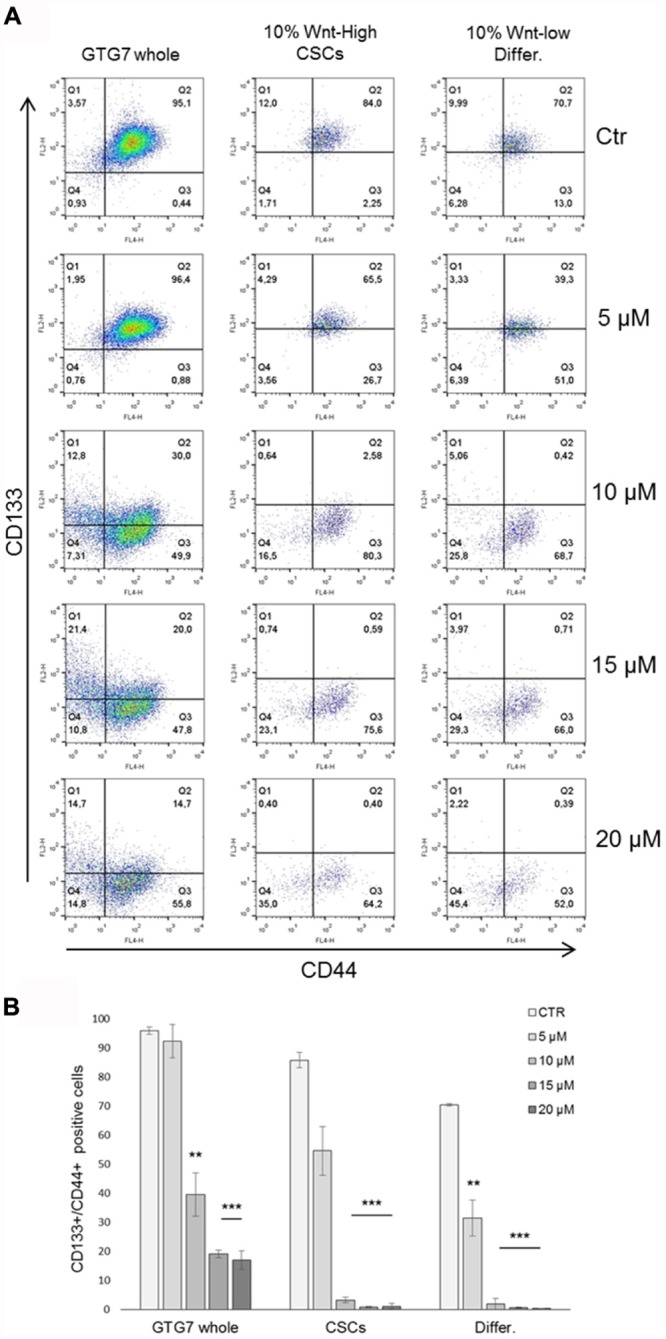
Representative FACS analysis of CD133-PE/CD44-APC double stained GTG7 treated with Rimonabant at indicated doses for 18 h. Analysis was performed on whole population (left panels) and gating on about 10% of Wnt^high^ cells and on about 10% Wnt^low^ cells (middle and right panels, respectively). Panels in **(A)** are representative and in **(B)** data are expressed as mean ± SD of at least three independent experiments in duplicate (^∗∗^*p* < 0.01, ^∗∗∗^*p* < 0.005 vs. control).

GTG7 is primary CRC stem cell line, stable transfected with Wnt-TOP-GFP reporter. Since Wnt activity correlates with cancer stemness (Vermeulen et al., 2010), in this culture it is possible to distinguish between CSCs (identified as TOP-GFP^high^ or Wnt^high^) or differentiated tumor cells (TOP-GFP^low^ or Wnt^low^), thereby taking into account the tumor heterogeneity. To evaluate Rimonabant-mediated cell death induction in CSCs, caspase-3 activity was measured with FACS-based CaspGlow active staining kit, gating on about 10% of TOP-GFP^high^ (CSCs) and, in same experiment, on about 10% TOP-GFP^low^ (differentiated tumor cells). FACS analysis of GTG7 cells treated for 24 h with Rimonabant (5–20 μM) revealed that the compound significantly activates caspase-3 both in Wnt^low^ differentiated tumor cells (in a more pronounced manner) and in Wnt^high^ CSCs, in a dose-dependent manner, starting from 10 μM (**Figures 3A,B**). This result reveals for the first time the ability of Rimonabant to induce cell death in high tumorigenic primary colorectal CSC line that, as previously reported, is chemoresistant to the commonly used chemodrugs (see Colak et al., 2014, where GTG7 are labeled as Co100 cells). Furthermore, the conspicuous DNA fragmentation evidenced after 24 h of treatment, supports a Rimonabant-mediated cell death induction in GTG7 cell line (**Figure 3C**). FACS-based analysis of Wnt activity in GTG7 also revealed that Rimonabant, in agreement with caspase-3 activation trend, significantly reduces Wnt activation in a dose-dependent manner, starting from 10 μM (**Figure 3A**). Wnt activity reduction was indeed preceded by a strong reduction of CD133^+^/CD44^+^ double positive cells. As expected, after 18 h of treatments, 5 μM dose of Rimonabant failed to reduce CD133^+^/CD44^+^ cells, while 10, 15, and 20 μM doses significantly reduce them, in both differentiated tumor cells and CSCs (**Figures 4A,B**).

**FIGURE 5:**
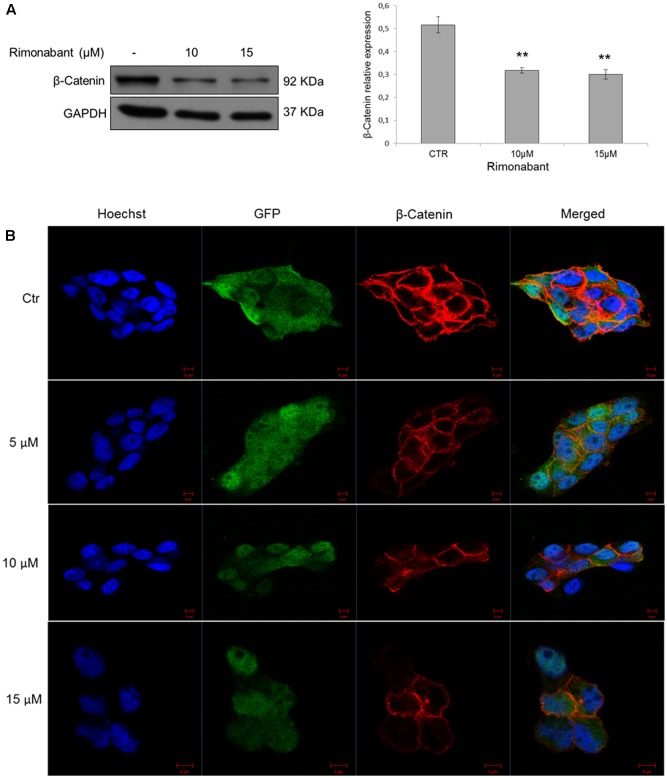
**(A)** Representative Western blot analysis (left) of β-Catenin in total protein lysates from GTG7 cells. Right, densitometric analysis of β-Catenin normalized vs. GAPDH, used as loading control. Results are expressed as means ± SD of 3 independent experiments performed at least in duplicate (^∗∗^*p* < 0.01). **(B)** Confocal microscopy analysis of β-Catenin (red) in GTG7 cells treated with Rimonabant at indicated doses for 18 h. Nuclei were stained with Hoechst (blue fluorescence); GFP (green); Scale bar = 5 μm.

Moreover, confocal microscopy analysis was performed on GTG7 to assess β-Catenin expression after treatments for 18 h with Rimonabant. According to previous observations, 10 and 15 μM doses of Rimonabant clearly reduces global β-Catenin expression, together with GFP intensity (**Figure 5B**). Western blot analysis, substantially confirmed β-Catenin expression reduction after treatment with active concentrations of 10 and 15 μM (**Figure 5A**).

### Rimonabant Shorten CSCs Survival in Long-term Cultures

**FIGURE 6:**
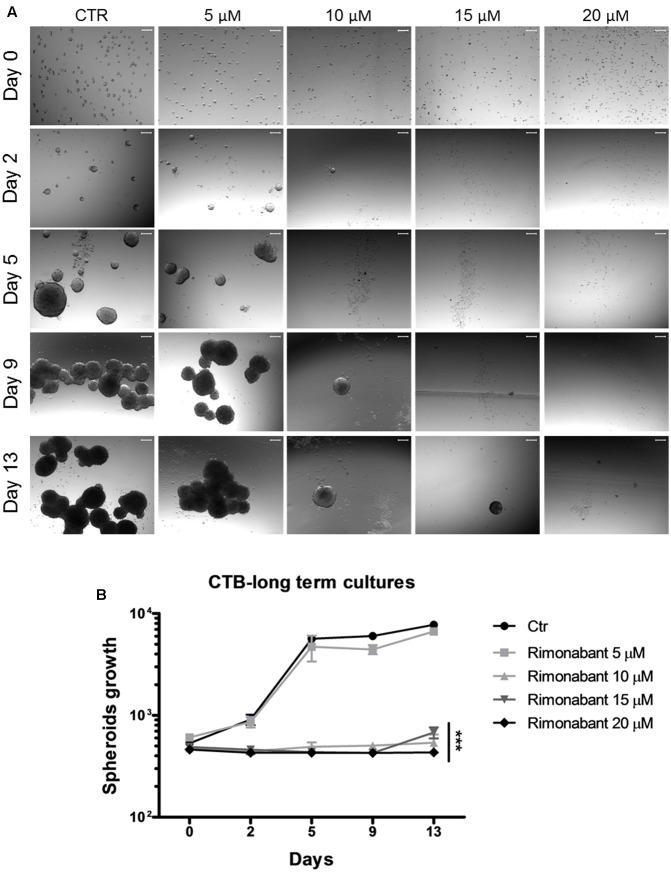
**(A)** Representative images of GTG7 clones after 24 h of treatment with Rimonabant (or vehicle control) starting from day 0 (corresponding to the end of treatments) and until day 13. Growth of spheroidal clones was monitored in three independent experiments in triplicate. Scale bar = 100 μm. **(B)** Cell Titer Blue (CTB) colorimetric assay in GTG7 treated for 24 h, as described. Data are expressed as mean ± SD of at least three independent experiments in triplicate (^∗∗∗^*p* < 0.005 vs. control).

Given their chemoresistance and high tumorigenicity, CSCs are substantially associated to tumor recurrence (Colak and Medema, 2014). Thus, development of novel strategies to overcome CSCs long-term survival and spreading is essential for an improved management of CRC. In the light of obtained results, Rimonabant effects on GTG7 long-term survival was analyzed. To this aim, GTG7 were treated with dose range 5–20 μM for 24 h. At the end of treatments, Rimonabant was removed from culture medium, the cells from each replicate were collected and seeded as spheroids in non-adherent conditions. Through Cell Titer Blue colorimetric assay, viability of spheroids was monitored starting from day 0 (which coincides with the end of treatments) for total 13 days. As shown in **Figures 6A,B**, Rimonabant strongly reduces GTG7 survival: with 10 and 15 μM doses, the appearance of vital clones were detected only after 9 days. After 13 days, at 20 μM dose, vital clones were not identified, probably suggesting an excessive toxicity of this dose. Substantially, reduction of GTG7 survival in long-term cultures supports Rimonabant ability of cancer stemness control.

### Rimonabant Does Not Improve Oxaliplatin and 5-Fluorouracil Effects in GTG7 Cells

**FIGURE 7:**
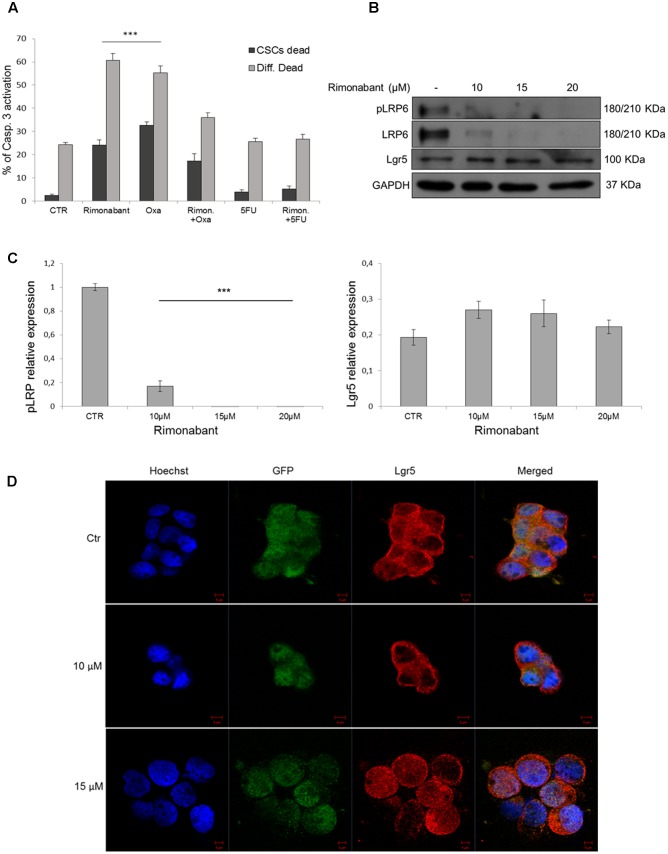
**(A)** Caspase 3 activation in GTG7 cells treated with single or combined compounds Rimonabant 10 μM, 5FU 25 μM, Oxaliplatin 25 μM or vehicle alone. Caspase 3 (PE quantification) was analyzed gating on about 10% of TOP-GFP^high^ (Wnt^high^) cells, corresponding to CSCs, and on about 10% TOP-GFP^low^ (Wnt^low^) cells, corresponding to differentiated tumor cells. Data are expressed as mean ± SD of at least three independent experiments in duplicate (^∗∗∗^*p* < 0.005 vs. control). **(B)** Representative Western blot analysis of Lgr5, LRP6 and phosphorylated (Ser1490) LRP6. GAPDH was used as loading control. **(C)** The histogram on the left represents the densitometric analysis of phospho-LRP6 expressed as fold change of the total LRP6 amount and normalized vs. GAPDH. Lgr5 expression (right histogram) was normalized vs. GAPDH. (^∗∗∗^*p* < 0.005 vs. control). **(D)** Confocal microscopy analysis of Lgr5 (red fluorescence) in GTG7 cells treated with Rimonabant at indicated doses for 18 h. Nuclei were stained with Hoechst (blue fluorescence); GFP (green); Scale bar = 5 μm.

Previous results obtained in HCT116 cell line, showed a synergistic interaction between Rimonabant and 5FU. Since CSCs are highly chemoresistant, FACS analysis of caspase-3 activation was performed in GTG7, on Wnt^high^ CSCs and Wnt^low^ subpopulations. To this aim, GTG7 were pre-treated for 6 h with Rimonabant (10 μM). At the end of pre-treatments, Oxaliplatin or 5FU (25 μM) were added for total 24 h. As shown, at these concentrations Rimonabant reduced the efficacy of Oxaliplatin and produced a slight but non-significant improvement of 5FU efficacy (**Figure 7A**). This result could suggests an antagonistic interaction, particularly with Oxaliplatin, at least for doses and time points analyzed.

As in HCT116 cells, we then analyzed Lgr5 expression in CSCs. In GTG7 Rimonabant treatments did not reduces Lgr5 expression (**Figures 7B,C**, right histogram). Confocal microscopy analysis substantially confirmed western blot results and revealed that after treatment with Rimonabant 10 μM, Lgr5 switch its localization predominantly to plasma membrane. Similar effect was not reported with 15 μM dose, where a more diffused signal was detected (**Figure 7D**). Lgr5 is a rodopsine-like GPCRs family member able to potentiate Wnt/β-Catenin signaling, through binding of its ligands R-Spondins. However, several authors reported that ligand binding is not associated with canonical GPCR signal (Carmon et al., 2011). The role of Lgr5 subcellular localization and trafficking is still an open question. Emerging evidence suggested that in the absence of endogenous ligands, Lgr5 is constitutively internalized through a clathrin-dependent mechanism that drive it to *trans*-Golgi network (Snyder et al., 2013). Even though pharmacological inhibition of Lgr5 internalization inhibits cell fitness *in vivo* (Snyder et al., 2017), it was reported that in presence of exogenous ligands, R-Spondins and Wnt3a, Lgr5 interacts with LRP6 receptor, forming a complex with Frizzled receptors and activate Wnt signal through phosphorylation of Serine 1490 residue of LRP6 (Carmon et al., 2011, 2012). To exclude this hypothesis we analyzed LRP6 phosphorylation (Ser1490) and, according to our previous observation, phosphorylated and total form of LRP6 result clearly abolished after treatments with Rimonabant (**Figures 7B,C**, left histogram). The different effect of Rimonabant 15 μM on Lgr5 localization remains to be clarified.

### Rimonabant Shows No Toxicity against Normal Colon Human Organoids

Encouraging results obtained in primary colon CSCs, strongly suggest that Rimonabant could be a novel lead compound in the treatment of high malignant CRC. Since an ideal therapeutic strategy consists of selective compounds toward cancer cells, with lowest toxicity against healthy tissues, evaluation of Rimonabant effects on normal colon was performed in wild type normal colon human organoids cultures.

**FIGURE 8:**
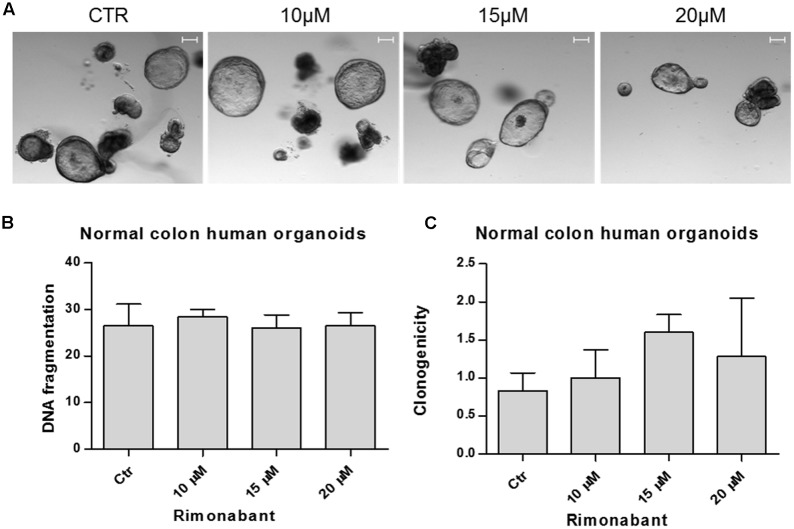
**(A)** Representative images of normal colon human organoids cultured in matrigel and treated for 24 h with Rimonabant or vehicle alone at the indicated doses. Scale bar = 100 μm. **(B)** Analysis of DNA fragmentation amount in PI-stained cells derived from normal colon human organoids, treated for 24 h with Rimonabant or vehicle alone at the indicated doses. **(C)** Analysis of normal colon human organoids clonogenicity after treatments with Rimonabant or vehicle alone at the indicated doses. Results represents the ratio of number of clones on day 7 vs. number of clones on day 0 (corresponding to the end of treatments). Data are expressed as mean ± SD of two independent experiments.

To this aim, normal colon organoids derived from patients were cultured in matrigel and treated for 24 h with Rimonabant used at the most active concentration in GTG7 (positive control used for experimental settings are showed in **Supplementary Figure S3**). At the end of treatments (**Figure 8A**), for each replicate matrigel was destroyed and removed. Crypts were collected, disaggregated and stained with PI for FACS evaluation of DNA fragmentation status. Surprisingly, FACS analysis of cells derived from Rimonabant treated organoids showed the same percent of fragmented DNA found in control organoids, also at 20 μM dose, suspected of extreme toxicity against GTG7 (**Figure 8B**). Finally, we performed clonogenic assay on normal colon organoids. After 24 h of treatments at indicated doses, Rimonabant was removed, crypt were collected, diluted and seeded again in new matrigel. Crypts were counted on day 0 (corresponding to end of treatments) and after 7 days. The clones number on day 7/clones number on day 0 ratio indicates that Rimonabant do not reduces clonogenicity of wild type normal colon organoids (**Figure 8C**).

## Discussion

Despite in the last decade the improvement of colon cancer management increased patient’s life expectancy, the occurrence of high malignancy forms and the progression toward metastatic and drug resistant phenotypes, remain a still open challenge to contrast disease relapses. Thus, discovery and development of new drugs aimed to improve therapeutic protocol for advanced malignancy are urgently needed.

It has become clear that CRC is a heterogeneous disease, both at intratumoral and intertumoral level (Punt et al., 2016). This increases difficulties in its management and, therefore, development of personalized medicine protocols based on molecular subtype could be the more advisable solution for an ideal therapy. Tumor heterogeneity implies that only a small subset of tumor cells retains tumorigenic ability, while a largest part differentiate in tumor cells that lost the ability to initiate cancer. The so-called CSCs, are certainly associated to tumor recurrence given their high chemoresistance to a large part of chemotherapeutics (Colak and Medema, 2014; Zhao, 2016).

Recently, Guinney et al. (2015), proposed a novel gene expression–based subtyping classification system for CRC. They identified four Consensus Molecular Subtypes (CMSs), with different signatures. CMS2 (canonical) is the largest subgroup (about 37%) presenting Myc and Wnt pathway hyperactivation. This result confirmed that Wnt/β-Catenin pathway plays a pivotal role in the onset and progression of CRC and the study of its intricate regulation is now emerging as a promising field to identify potential target of intervention for CRC treatment.

Wnt and c-Myc hyperactivity are undisputable markers of colon CSCs (Zeuner et al., 2014). Understanding of plastic and dynamic regulation surrounding cancer stem cells intra-clonal heterogeneity, raised the need to develop *in vitro* models that faithfully reflect patient’s tumors, aimed to improve personalized medicine through the knowledge of CSCs biology.

For decades, despite some unresolved disputes, endocannabinoid system modulators have gained increasing attention not only for their effects against cancer-related symptoms (such as anti-inflammatory, pain relief or anti-emetic action), but also as anti-cancer agents in many tumor types, including colon cancer (Maida and Daeninck, 2016; Velasco et al., 2016). Many reports indicate that cannabinoid compounds are able to reduce progression of CRC *in vivo* in the azoxymethane (AOM) induced ACF model in mice and to improve the efficacy of chemotherapeutic drugs used in the clinical practice (Izzo et al., 2008; Gustafsson et al., 2009; Santoro et al., 2009; Gazzerro et al., 2010). Moreover, in CRC cells, high levels of cannabinoids induced up-regulation of CB1-receptor through co-localization of PPARγ and RXRα at its promoting region, suggesting a potential epigenetic modulation in cannabinoid-mediated anti-tumor effects (Proto et al., 2012).

DeMorrow et al. (2008) reported that anandamide (AEA), a CB1 agonist, exerts anti-tumor effects in cholangiocarcinoma model, through activation of the non-canonical Wnt pathway. Moreover, in human breast cancer cells, a stable analogue of endogenous anandamide (Met-F-AEA) inhibits β-Catenin transcriptional activity on TCF/LEF responsive elements (Laezza et al., 2012). More interesting, some reports demonstrated the ability of cannabinoids to inhibit gliomagenesis, targeting glioma stem cells (GSCs) (Aguado et al., 2007; Nabissi et al., 2015), and spheroid formation in prostate cancer stem cells (Sharma et al., 2014).

Previous works from our group reported that Rimonabant, an antagonist/inverse agonist of CB1 receptor, is able to exert anti-tumor action in different cancer models (Malfitano et al., 2012; Ciaglia et al., 2015). Of note, in DLD1 CRC cells, Rimonabant produced mitotic catastrophe and modulated the expression of Cyclin B1, PARP-1, Aurora B and phosphorylated p38/MAPK and Chk1, improving the efficacy of Oxaliplatin (Santoro et al., 2009; Gazzerro et al., 2010).

In our recent work, we demonstrated that Rimonabant inhibits Wnt pathway activation and β-Catenin nuclear translocation both *in vitro* and *in vivo* models of CRC. The Rimonabant-mediated inhibition of TCF/LEF transcription factors allow to reduction of COX-2, Cyclin D1 and c-Myc expression. Moreover, through computational studies, we identified potential protein targets of Rimonabant and, among others, p300/Kat3b. We then demonstrated that Rimonabant was able to inhibits p300-HAT activity, suggesting that Wnt inhibition could be ascribable to downstream epigenetic regulation (Proto et al., 2017). In this work we tested the hypothesis that Rimonabant could control colon cancer stemness. In line with previous results, we found that Rimonabant reduces CD133+/CD44+ population in DLD1 and HCT116 cells, where it also downregulates Lgr5 expression. Moreover, in HCT116 cells, Rimonabant was able to produce a strong synergistic interaction with 5-Fluorouracil, to date one of the widely used drugs in CRC therapies. In DLD1 cells, as well as in GTG7 cells, we did not found downregulation of Lgr5 protein expression. Beyond its role as CSCs marker, Lgr5 receptor is strongly involved in negative feedback control of Wnt receptors turnover. Among R-Spondins (RSPO) ligands family, RSPO2 binding to Lgr5 regulates receptor turnover through stabilization of tumor suppressors ZNFR3 and RNF43 membrane E3 ligases, that mediate ubiquitination and consequent degradation of LRP6/5 receptors (Hao et al., 2016). As widely reported, the response to almost all chemodrugs is highly influenced by genotypes. Throughout RSPO2-Lgr5 signaling in CRC cells, several gene mutations were found. Both HCT116 and DLD1 cells, express low levels of RSPO2, due to its promoter methylation (Wu et al., 2014). Moreover, in HCT116 cells homozygous RNF43 mutation has been reported. Interestingly, the growth of RNF43 mutant tumors, such as HCT116, seem to be dependent on paracrine Wnt signal and, unlike to APC mutant such as DLD1, those tumors are sensitive to inhibitors of Wnt ligand secretion (Koo et al., 2012; Hao et al., 2016). We previously found a Rimonabant-mediated direct inhibition of Wnt/β-Catenin pathway in RNF43 and β-Catenin HCT116 mutant cells. On the other hand, in APC mutant DLD1 cells, we reported an indirect inhibition of Wnt signaling, substantially ascribable to p300-HAT inhibition (Proto et al., 2017). Finally, the Lgr5 splicing variants reported in CRCs, seem to influence also CSCs chemoresistance (Osawa et al., 2016).

Vermeulen et al. (2010) developed a 3D *in vitro* model of primary CSCs in which it is possible to take into account the tumor heterogeneity, monitoring heterogeneous Wnt activity. Here, in this model we found that Rimonabant was able to control cancer stemness, through activation of caspase 3, and then induction of cell death, in both differentiated tumor cells (characterized by Wnt-low activity) and in CSCs (defined as Wnt-high active cells). Together with the shorter survival of CSCs observed in long-term cultures and the reduction of β-Catenin expression and CD133+/CD44+ population, this result strongly supported Rimonabant ability to control colon cancer stem cells and their plasticity. Unfortunately, in primary CSCs Rimonabant did not ameliorate nor Oxaliplatin neither 5-Fluorouracil effects. Highest chemoresistance of CSCs seems to be ascribable to several mechanisms such as the high activity of drug transporters (e.g., MRP5) and to slowed cell cycling (Dean et al., 2005). Interestingly, Todaro et al. (2007) demonstrated that IL-4 inhibition enhances colon CSCs response to Oxaliplatin and 5FU. It was previously reported that, compared to differentiated cells, CSCs display a higher chemoresistance because of decreased mitochondrial priming and that BCL2, BCLXL or BCLW overexpression, increase chemotherapy resistance. However, in the same models we used in this work, BCL2-specific inhibitors failed to sensitize CSCs to chemodrugs such as Oxaliplatin (see Colak et al., 2014, where GTG7 are labeled as Co100 cells). In HCT116 cells, we previously found that Rimonabant induces apoptosis through reduction of BCL2 expression (Proto et al., 2017). Thus, this observation could explain the different results from combined treatments, observed in HCT116 and GTG7, where Rimonbant failed to sensitize the cells to Oxaliplatin and 5FU.

Mechanistic insights about Rimonabant effects and Endocannabinoid System regulation in GTG7, CSCs expressing high levels of CB1 receptor (see **Supplementary Figure S2**), need to be improved. Lgr5 is involved in metastasis dissemination and CSCs plasticity, more than in tumor maintenance (Melo et al., 2017). Its expression was not affected by Rimonabant treatments but, on the other hand, its localization seems to be modified. Lgr5 is constitutively internalized through a clathrin-dependent mechanism (Snyder et al., 2013). The anti-proliferative effect of Rimonabant in breast cancer cells occurs through a CB1-lipid raft/caveolae-mediated mechanism (Sarnataro et al., 2006). This evidence, together with observation of complete abolishment of LRP6 expression, allows us to speculate that an unknown mechanism occurs through plasma membrane. Moreover, the different regulation of Lgr5 receptor, observed in HCT116, DLD1 and GTG7 cells carrying different genotypes, suggests that Rimonabant could affect the intricate feedback regulation of Wnt ligands and receptors also through plasma membrane. Therefore, further studies are required to dissect the precise events underling Rimonabant-mediated effects.

Since Wnt/β-Catenin pathway regulates both normal and cancer tissues homeostasis, safely eradication of CSCs and, generally, of cancer cells, need to be improved by discovery of selective drugs. In recent years, development of *ex vivo* cultures “organoids,” in which material from patients can be cultured *in vitro*, retaining both architecture and heterogeneity of original tissue, pioneered the better understanding of both tumor biology and normal tissue homeostasis. These models are currently used for a wide spectrum of application, such as personalized medicine, living biobanks, genetic analysis, but also drug discovery (Leushacke and Barker, 2014; Ohta and Sato, 2014). In our previous work, we reported the anti-proliferative effect of Rimonabant in glioma cell line and primary cells from patients, without significant toxicity in Normal Human Astrocyte (NHA), thus demonstrating moderate selectivity toward glioma cells, *in vitro* (Ciaglia et al., 2015). Here, for the first time, we demonstrated that in normal colon human organoids (wild type organoids that represent a high fidelity surrogate of normal colon epithelium) Rimonabant did not shows toxicity or clonogenicity reduction in normal cells, also at higher doses. To date, many reports tried to elucidate distribution of receptors, ligands and enzymes of endocannabinoid system in the gut. While CB2 receptor shows relevant role in mucosal immunity, being expressed prevalently in intestinal macrophages, CB1 receptor seems to be expressed in smooth muscle, submucosal myenteric plexus and normal human colon (Wright et al., 2005). The intricate regulation of this system seems to be contest- and cell-specific and may totally differ depending on patho-physiological conditions, but the exact regulation must still be clarified to optimize clinical use of specific cannabinoid compounds. Some authors reported that acute administration of Rimonabant (3 and 5.6 mg/kg dose), increases gastrointestinal motility, diarrhea and nausea, in a dose-dependent manner. However, Carai et al. (2004) demonstrated that in mice, after initial induction of intestinal peristalsis, tolerance mechanisms occurred (Carai et al., 2004; Izzo and Sharkey, 2010). Of course, since normal colon organoids contain all cell type of intestinal crypt and villus compartments (e.g., Lgr5+ niche stem cells, goblet cells, Paneth cells etc.) (Leushacke and Barker, 2014), with these preliminary results we cannot establish if Rimonabant influences differentiation or, generally, homeostasis of normal epithelium, in cell-specific manner. Thus, additional experiments would be needed to deepen this aspect.

Substantially, these promising results could candidate Rimonabant as a novel lead compound for CRC treatments, and could help its optimization as chemotherapic agent, also taking advantage of information obtained in previous clinical studies as anti-obesity drug.

## Author Contributions

DF designed and conducted research, analyzed and interpreted data, wrote the paper. PR designed and conducted research. MP, CP, SF, and SA performed research and analyzed data. JM designed research and gave substantial contribution to critical analysis and interpretation of data. MB and PG designed and supervised the project in its entirety, provided financial support, wrote and critically reviewed the paper. All authors approved the final version of the manuscript.

## Conflict of Interest Statement

The authors declare that the research was conducted in the absence of any commercial or financial relationships that could be construed as a potential conflict of interest.

## Supplementary Material

**FIGURE S1** | **(A)** Representative Western blot analysis of CD44, CD133 and Lgr5 in total protein lysates from DLD1 cells. GAPDH was used as loading control. Right histogram represents densitometric analysis of CD44, CD133 and Lgr5 normalized vs. GAPDH. **(B)** Representative Western blot analysis of β-Catenin expression in DLD1 and HCT116 cells treated with 10 μM of Rimonabant for 48 h. Right histogram represents densitometric analysis of β-Catenin normalized vs. GAPDH. Results are expressed as means ± SD of 3 independent experiments performed at least in duplicate (^∗∗^*p* < 0.01, ^∗∗∗^*p* < 0.001 vs. control).

**FIGURE S2** | Representative Western Blot of CB1 receptor in GTG7 and in HCT116. GAPDH was used as loading control.

**FIGURE S3** | **(A)** Representative images of wild type organoids treated with 50 μM dose of Rimonabant (two replicates), the highest concentration used as positive control. **(B)** Representative images of GTG7 spheroids cultured in matrigel, treated for 24 h with Rimonabant at indicated concentrations. Scale bar = 100 μm.

**TABLE S1** | Combination Index (CI) values obtained from Compusyn analysis of HCT116 cells treated for 24 h with Rimonabant and 5FU (constant molar ratio 1:2.5). Data are representative of at least three independent experiments performed in triplicate with similar results.

**TABLE S2** | Dose Reduction Index calculated by Compusyn software for Rimonabant (0.16 – 10 μM) and 5FU (0.4 – 25 μM) combined at constant molar ratio 1:2.5. Data are representative of at least three independent experiments performed in triplicate with similar results.

**TABLE S3** | IC50 values (μM) obtained by cell viability assays for GTG7 cells treated for 24 h with Rimonabant and HCT116 cells treated for 24 h with Rimonabant or 5FU. Data are expressed as mean ± SD values of three independent experiments, each made at least in triplicate.
