# Supplementary figures and images for: Rimonabant Kills Colon Cancer Stem Cells without Inducing Toxicity in Normal Colon Organoids
Source: Front Pharmacol. 2018 Jan 4;8:949. doi: 10.3389/fphar.2017.00949 (PMC5758598; doi:10.3389/fphar.2017.00949)

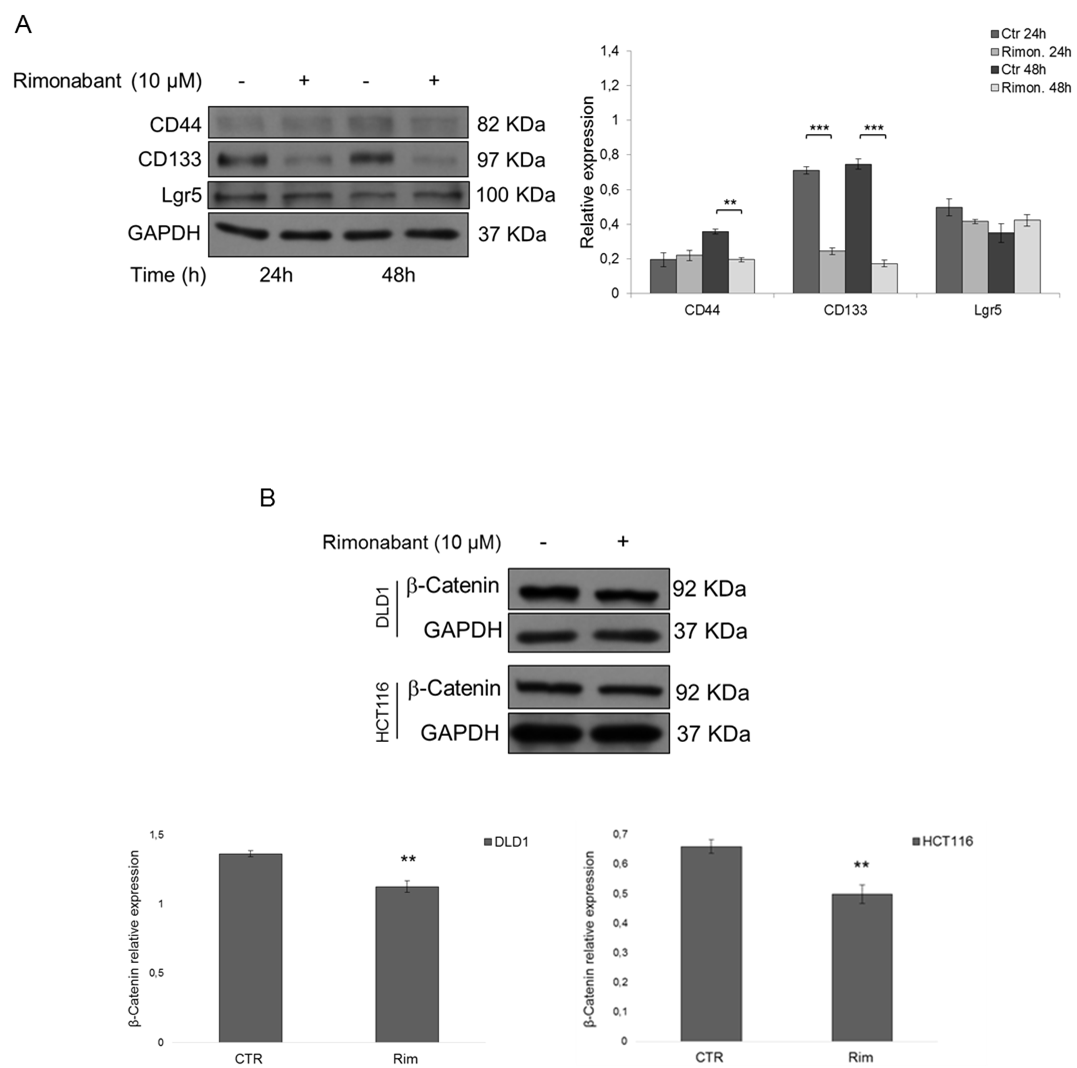

Supplement: Supplementary file 1 [file Image_1.TIF]

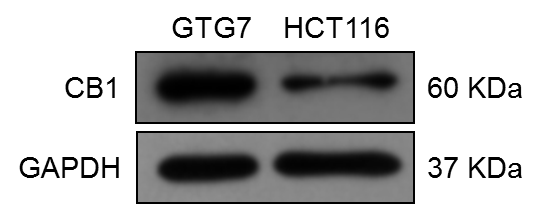

Supplement: Supplementary file 2 [file Image_2.TIF]

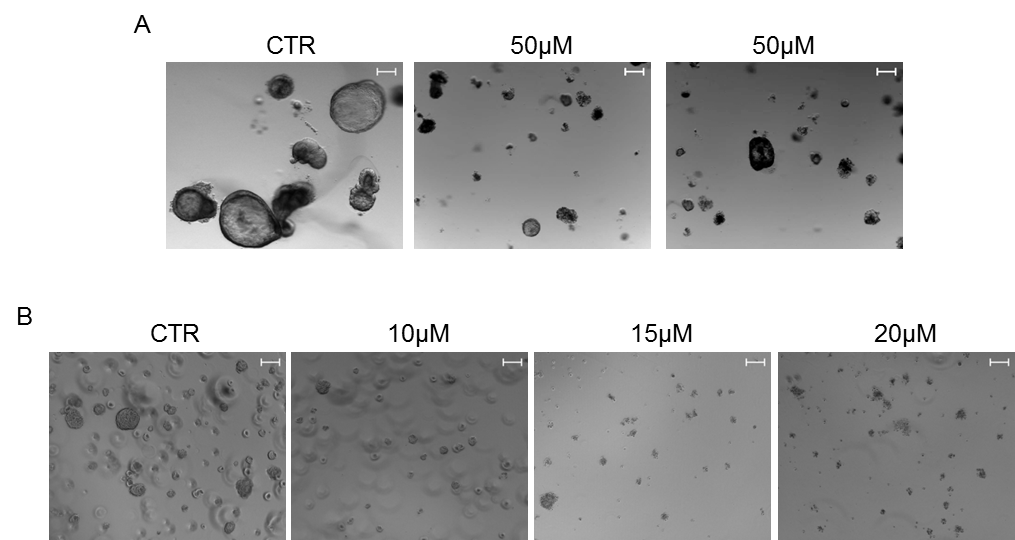

Supplement: Supplementary file 3 [file Image_3.TIF]
