# Supplementary material for: Rimonabant Kills Colon Cancer Stem Cells without Inducing Toxicity in Normal Colon Organoids
Source: Front Pharmacol. 2018 Jan 4;8:949. doi: 10.3389/fphar.2017.00949 (PMC5758598; doi:10.3389/fphar.2017.00949)
Supplement: Supplementary file 4 [file Table_1.PDF]

Supplementary Table 1

CI values for actual experimental points:

Total Dose	Fa	CI Value
0.56	0.52333	0.03933
1.085	0.53879	0.07336
2.1875	0.62543	0.12445
4.375	0.68469	0.22662
8.75	0.84656	0.35278
17.5	0.91927	0.59444
35.0	0.91428	1.20721
