# Supplementary material for: Rimonabant Kills Colon Cancer Stem Cells without Inducing Toxicity in Normal Colon Organoids
Source: Front Pharmacol. 2018 Jan 4;8:949. doi: 10.3389/fphar.2017.00949 (PMC5758598; doi:10.3389/fphar.2017.00949)
Supplement: Supplementary file 5 [file Table_2.PDF]

Supplementary Table 2

DRI values calculated at experimental points

Fa	Dose SR	Dose 5FU	DRI SR	DRI 5FU
0.52333	4.89509	60.1832	30.5943	150.458
0.53879	4.96604	70.8897	16.0195	91.4705
0.62543	5.39462	181.823	8.63139	116.367
0.68469	5.73331	363.537	4.58665	116.332
0.84656	7.11626	4249.87	2.84650	679.979
0.91927	8.41742	28719.5	1.68348	2297.56
0.91428	8.29070	24166.6	0.82907	966.664
