# Supplementary material for: Rimonabant Kills Colon Cancer Stem Cells without Inducing Toxicity in Normal Colon Organoids
Source: Front Pharmacol. 2018 Jan 4;8:949. doi: 10.3389/fphar.2017.00949 (PMC5758598; doi:10.3389/fphar.2017.00949)
Supplement: Supplementary file 6 [file Table_3.PDF]

Supplementary Table 3

	HCT116	GTG7
**Rimonabant**	2,93 ± 0,11	7,51 ± 8,67
**5FU**	16,61 ± 6,96	-
